# Targeting ZC3H11A elicits immunogenic cancer cell death through augmentation of antigen presentation and interferon response

**DOI:** 10.1016/j.omtn.2024.102361

**Published:** 2024-10-21

**Authors:** Arwa Ali, Paola Contreras, Mahmoud Darweesh, Leif Andersson, Chuan Jin, Magnus Essand, Di Yu

**Affiliations:** 1 Department of Immunology, Genetics, and Pathology, Uppsala University, 751 85 Uppsala, Sweden; 2 Department of Medical Biochemistry and Microbiology, Uppsala University, 751 23 Uppsala, Sweden; 3 Department of Microbiology and Immunology, Faculty of Pharmacy, Al-Azhr University, Assiut 71526, Egypt; 4 Immunolgy Laboratory, Natural and Medical Sciences Research Centre (NMSRC), University of Nizwa, PO.Box:33, P.C. 616 Nizwa, Oman; 5 Department of Veterinary Integrative Biosciences, Texas A&M University, College Station, TX 77843, USA

**Keywords:** MT: Oligonucleotides: Therapies and Applications, ZC3H11A, ASOs, antigen presentation, IFN response, immunogenic apoptosis, cancer, immunogenic cell death, ICD

## Abstract

Zinc finger CCCH containing 11A (ZC3H11A) is a stress-induced protein that is upregulated in various conditions such as heat shock and virus infection. It has also been reported to be upregulated in certain cancers. The aim of this study was to evaluate the feasibility of targeting ZC3H11A as a therapeutic approach for cancer treatment, using nuclease-resistant, affinity-enhanced antisense oligonucleotide (ASO). An ASO targeting ZC3H11A was validated and evaluated *in vitro* and in the B16 melanoma model *in vivo*. Antigen presentation, interferon response, cell proliferation, and apoptosis were transcriptionally affected. These findings were validated on the protein level by the upregulation of major histocompatibility complex class I (MHC class I), an increased secretion of interferon-β (IFN-β), and induction of apoptosis observed as upregulation of caspases and annexin V. Immunogenic features of the induced apoptosis were evidenced by the surface exposure of calreticulin (CRT) and the secretion of ATP leading to enhanced dendritic cell (DC) phagocytosis, maturation, and activation. Treatment with the ZC3H11A-targeted ASO had limited efficacy *in vivo*, while constitutive lentiviral shRNA knockdown of ZC3H11A in murine B16 melanoma cells and human HeLa cells led to reduced tumor growth with prolonged survival of mice, validating ZC3H11A as a relevant target for cancer therapy.

## Graphical abstract

**Figure:**
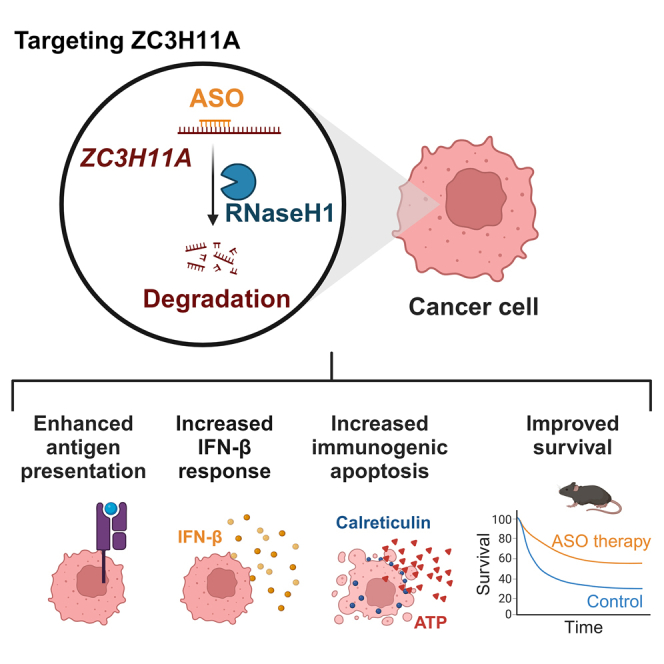

## Teaser

Yu and colleagues showed that targeting the stress-induced zinc finger protein ZC3H11A can be a potential cancer therapy. The authors utilized antisense oligonucleotides to knock down ZC3H11A in different cancer models and observed increased interferon response, antigen presentation, and immunogenic apoptosis, which are collectively crucial for boosting the immune response against cancer.

## Introduction

ZC3H11A is a zinc finger protein comprising three CCH-zinc finger motifs.^1^ The *ZC3H11A* gene serves as a host for ZBED6 (Zinc Finger BED-Type Containing 6), a transcription factor that represses the insulin-like growth factor 2 gene.^2^ ZC3H11A has been found to be a component of the transcription-export (TREX) complex, a conserved system for exporting mRNA from the nucleus to the cytoplasm.^3^^,^^4^^,^^5^^,^^6^ It has been reported that ZC3H11A is “hijacked” by viruses to export their mRNA from the nucleus, as evidenced by a reduction in viral replication upon knocking out ZC3H11A.^6^ Recent research has highlighted the importance of this protein in mouse embryogenesis, as it is involved in glycolysis and fatty acid metabolism.^7^ ZC3H11A has been shown to suppress NF-κB (nuclear factor κB) signaling by retaining IkBα (NF-κB inhibitor α), which is necessary for the negative feedback loop of this signaling pathway.^8^ ZC3H11A has been identified as a stress-induced protein that is highly upregulated in stress conditions such as virus infection and heat shock.^6^ In the context of cancer, several studies have reported high levels of ZC3H11A in cancer tissues compared to normal tissues.^9^^,^^10^^,^^11^^,^^12^^,^^13^ It is believed that the machinery of mRNA export is deregulated in cancer, leading to the export of RNA that encodes proteins involved in tumorigenesis.^14^ As such, ZC3H11A may contribute to the development of different types of cancer (including colorectal carcinoma) by participating in mRNA processing and nuclear exporting processes, which are critical for tumor progression.^9^ Moreover, in a melanoma study, it was found that the overexpression of ZC3H11A, CEP170, and NUCKS1 work collectively as competitive endogenous RNA to sequester microRNAs that potentially have a suppressive role in cancer progression and metastasis.^13^

The utilization of antisense oligonucleotides (ASOs) as a therapeutic agent holds great promise in the treatment of various diseases. To enhance the binding affinity and specificity with target mRNAs, as well as increase stability upon *in vivo* injection, chemical modifications can be incorporated into ASOs.^15^ These molecules can block translation through various mechanisms, such as RNase H-mediated cleavage when bound to mRNA.^16^ The US Food and Drug Administration has already approved ASOs for the treatment of conditions like hypercholesterolemia and muscular dystrophy.^17^ Although no ASO has yet been approved for cancer treatment, several ASOs are currently undergoing clinical trials.^18^ Our study aimed to investigate the impact of inhibiting the ZC3H11A gene in cancer, particularly melanoma and colorectal carcinoma, using ASO as a tool to evaluate ZC3H11A function and evaluate ZC3H11A ASOs as a potential therapeutic agent.

## Results

### Efficient knockdown of murine ZC3H11A by targeted ASO

**Figure 1:**
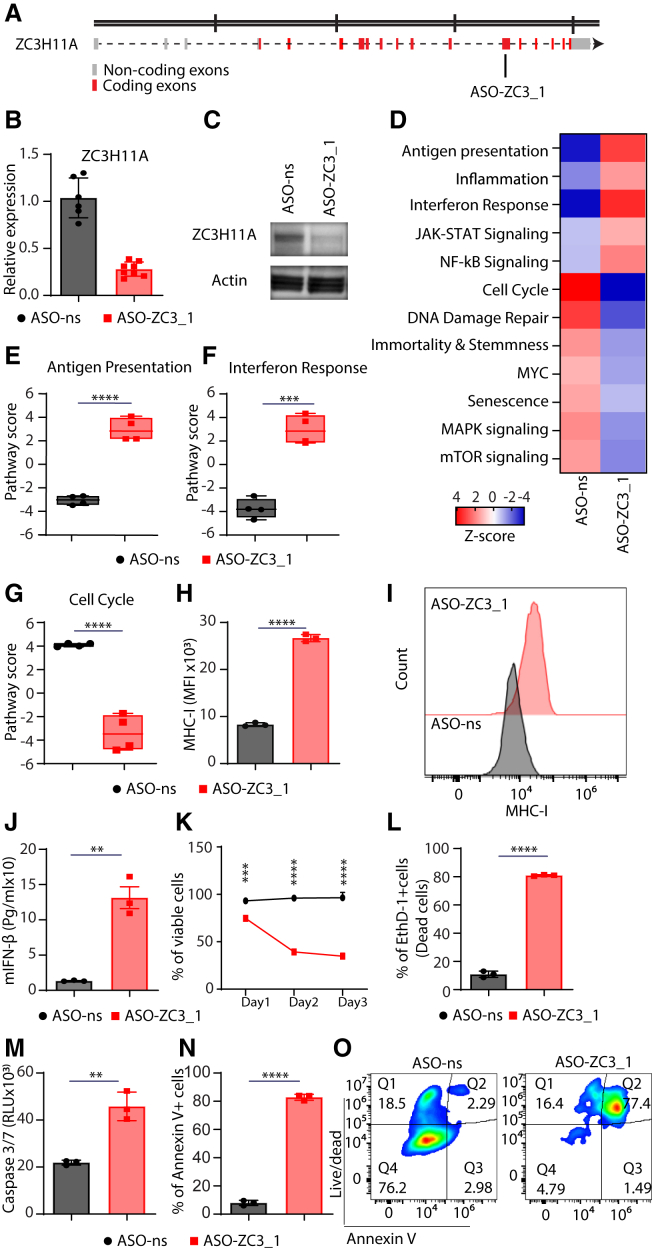
Knockdown of ZC3H11A by ASO results in enhanced antigen presentation, IFN response, and apoptosis in B16 melanoma cells (A) Schematic illustration showing the location of the ASO-ZC3_1 target within ZC3H11A. (B) The knockdown efficiency of ASO-ZC3_1 (10 nM) estimated at the RNA level by real-time qPCR; gene expression of ZC3H11A was normalized to HPRT and expressed relative to untreated cells (only media) (*n* = 6–8 replicates/group). (C) Western blots showing the knockdown efficiency of ZC3H11_A at the protein level after ASO-ns and ASO-ZC3_1 treatment, using actin as a reference protein. (D–G) Pathway signature scores determined by NanoString mRNA profiling (*n* = 4 replicates/group). (D) The heatmap shows the *Z* score of the different pathways that were upregulated or downregulated after treatment with ASO-ns and ASO-ZC3_1. Boxplots show the pathway scores of Ag presentation (E), IFN response (F), and cell cycle (G). (H and I) MHC class I expression determined by flow cytometry (*n* = 3 replicates/group), with representative histograms. (J) Analysis of IFN-β released by ELISA after ASO transfection and polyI:C (50 μg/mL) treatment of B16 cells (*n* = 3 replicates/group). (K) Line graph shows the percentage of viable cells of both ASO-ns and ASO-ZC3_1 transfected B16 over 4 consecutive days (*n* = 3 replicates/group). (L) Percentage of EthD-1^+^ cells (dead cells) (*n* = 3 replicates/group). (M) The caspase-3/-7 level (RLU) and (N and O) percentage of annexin V^+^ cells (*n* = 3 replicates/group), with representative plots. Error bars represent SDs, and the mean values were compared using an unpaired two-tailed t test. ∗∗*p* < 0.01; ∗∗∗*p* < 0.001; ∗∗∗∗*p* < 0.0001.

An ASO targeting ZC3H11A (ASO-ZC3_1) (Figure 1A) was designed and evaluated. To establish the most effective concentration for inducing substantial knockdown, we conducted transient transfection experiments on murine melanoma B16 cells employing various concentrations, ranging from 0.1 to 10 nM. Notably, significant knockdown efficiency became apparent at concentrations of 3 nM and higher (Figures S1A and 1B). Furthermore, the onset of knockdown was observed as early as 6 h post-treatment (Figure S1B). We also verified the knockdown effectiveness by QuantiGene Singleplex assay (Figure S1C). Validation at the protein level further reinforced these findings (Figure 1C). Expanding our investigations, we extended these observations to an additional murine melanoma (HCmel12) cell line and a mouse colorectal carcinoma (CT26) cell line, where ASO-ZC3_1 exhibited substantial knockdown efficiency on RNA (Figures S2A and S2B) and protein (Figure S2C) levels. Additionally, employing a second ASO, ASO-ZC3_2, targeting a distinct site within *ZC3H11A* (Figure S3A), we observed efficient knockdown in both B16 and CT26 cell lines (Figures S3B–S3D).

### ASO targeting of murine ZC3H11A (ASO-ZC3_1) enhances antigen presentation and type I interferon production and affects cell viability

We conducted NanoString RNA profiling of B16 melanoma cells that were transfected with ASO-ZC3_1 to determine the impact of ZC3H11A knockdown on pathway activity. Our analysis revealed significant changes in pathway signature scores (Figure 1D), with a marked shift in the signature scores of antigen (Ag) presentation (Figure 1E), and interferon (IFN) response (Figure 1F), which were drastically increased, while the score for the cell cycle was significantly reduced (Figure 1G). As verifications for the NanoString profiling, we performed cell culture experiments and found that the expression of MHC class I in the transfected cells was markedly increased after knockdown of ZC3H11A (Figures 1H and 1I). Moreover, when stimulated with polyI:C, we observed a significant increase in IFN-β secretion (Figure 1J) in the ASO-ZC3-treated B16, while the IFN-γ level remained unchanged (data not shown). Our data also showed a dramatic decrease in cell viability (Figure 1K), a significant increase in dead cells (Figure 1L), high levels of caspase-3/-7 (Figure 1M), and an increased percentage of annexin V^+^ cells (Figures 1N and 1O). These observations were also replicated in HCmel12 and CT26 cells (Figures S2D–S2Q). Additionally, ASO-ZC3_2 was found to affect the same pathways (Figures S3E–S3Q). Taken together, these results indicate knockdown of ZC3H11A enhanced Ag presentation, IFN response and promoted cell death.

### Knockdown of ZC3H11A by ASO induces immunogenic apoptosis

**Figure 2:**
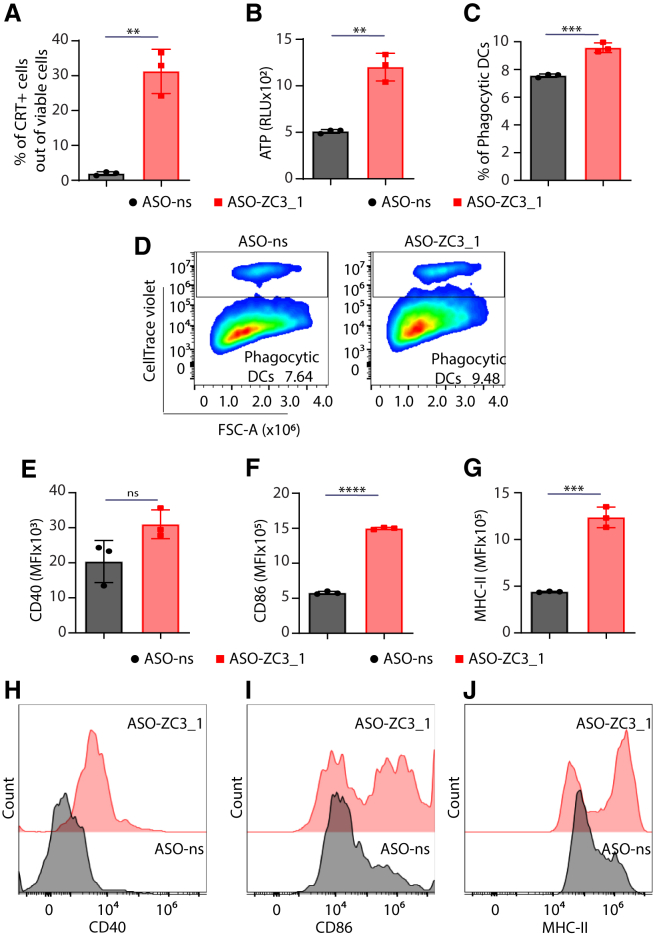
Knockdown of ZC3H11A by ASO induces immunogenic apoptosis in B16 melanoma cells (A) The percentage of calreticulin (CRT)^+^ cells analyzed out of viable cells by flow cytometry in B16 cells after 36 h of ASO transfection. (B) ATP levels (RLU) in the supernatant of ASO-transfected B16 cells. (C and D) The percentage of dendritic cells (DCs) that phagocytized ASOs transfected with B16 stained with CellTrace violet dye (CTV) and representative plots of flow cytometry. Mean fluorescence intensity (MFI) of DC activation and maturation markers (E) CD40, (F) CD86, and (G) MHC class II after co-culture of DCs with ASO-transfected B16 cells. (H–J) Representative histograms of the activation markers. *n* = 3 replicates/group for (A)–(C) and (E)–(G). Error bars represent SDs, and the mean values were compared using an unpaired two-tailed t test. ns, non-significant; ∗∗*p* < 0.01; ∗∗∗*p* < 0.001; ∗∗∗∗*p* < 0.0001.

We aimed to evaluate whether the apoptosis triggered by ASO-ZC3_1 shows immunogenic characteristics. To address this, we investigated specific markers associated with immunogenic cell death, focusing on calreticulin (CRT) exposure and ATP release. Our findings revealed a notable increase in both CRT exposure and ATP release when comparing ASO-ZC3_1-treated cells to those treated with nonsense ASO (ASO-ns) (Figures 2A and 2B). Further examinations demonstrated that this immunogenic apoptosis significantly strengthened the phagocytic activity of immature dendritic cells (imDCs) (Figures 2C and 2D) and contributed to their activation and maturation processes (Figures 2E–2J). Notably, both HCmel12 and CT26 cell lines exhibited amplified CRT exposure after ASO-ZC3_1 treatment (Figures S4A and S4B). Additionally, in HCmel12 cells, the induction of immunogenic apoptosis correlated with an increased phagocytosis of DCs (Figures S4C and S4D) and enhanced activation (Figures S4E–S4J). Furthermore, ASO-ZC3_2 treatment exhibited similar immunogenic cell death patterns, coupled with increased CRT exposure (Figures S5A and S5B) and DC phagocytosis in both B16 and CT26 cells (Figures S5C–S5F). It is worth noting that the activation of DCs was also observed specifically in B16 cells (Figures S5G–S5N). Taken together, these findings collectively highlight the immunogenic nature of the apoptosis induced by ASO-ZC3 across multiple cell lines, suggesting its potential in modulating immune responses.

### Targeting ZC3H11A inhibits tumor growth and improves mice survival

**Figure 3:**
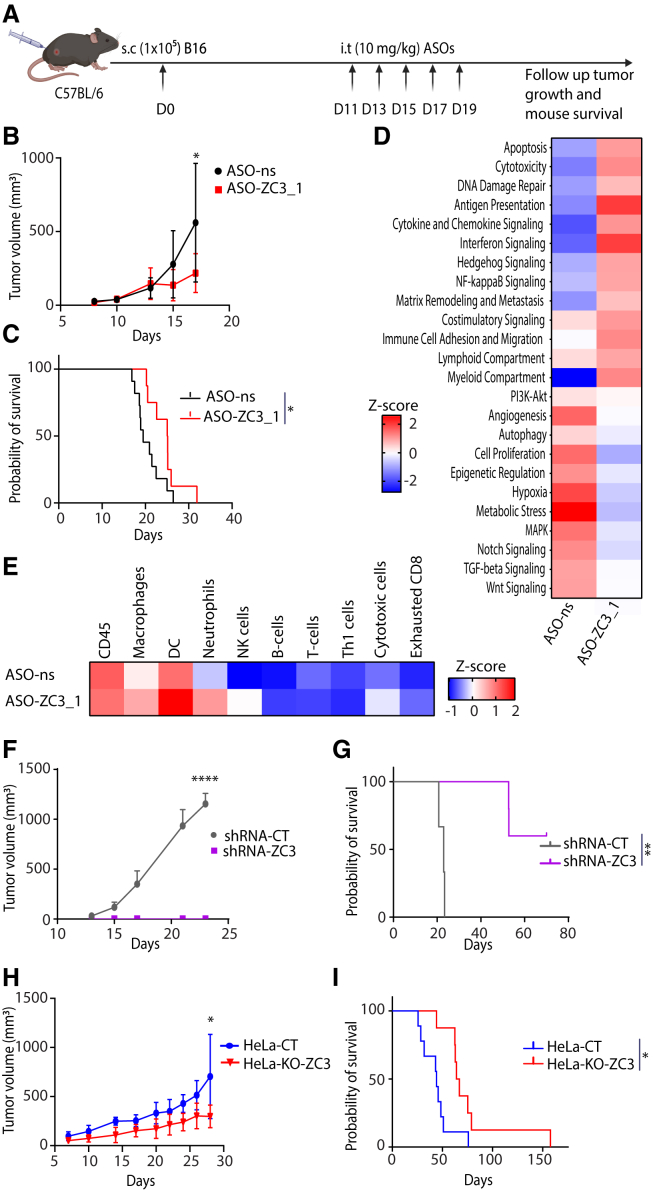
Knockdown or knockout of ZC3H11A slows tumor growth and improves mouse survival (A) The experimental setup for the therapeutic efficacy of ASO-ZC3_1 in the B16 model is depicted in a schematic illustration. Briefly, B16 melanoma cells were subcutaneously implanted into C57BL/6 mice on day 0. On day 11, ASO (10 mg/kg) combined with PEI was injected intratumorally five times, and mice were sacrificed at the humane endpoint. (B) Volume (mm^3^) of B16 tumors treated with either ASO-ns or ASO-ZC3_1. (C) The time-to-endpoint (TTE) was calculated to plot mouse survival (Kaplan-Meier curve) after treatment with ASO-ns and ASO-ZC3_1, as indicated (ASO-ns = 11 and ASO-ZC3_1 = 8). (D and E) The heatmap from NanoString shows the *Z* scores of (D) different pathways and (E) cell types that were upregulated or downregulated in dissected tumors after treatment with ASO-ns and ASO-ZC3_1 (ASO-ns = 3 and ASO-ZC3_1 = 3). (F) Tumor volume (mm^3^) of shRNA-CT (control) and shRNA-ZC3 (knockdown) B16 tumors after subcutaneous implantation into C57BL/6 mice (shRNA-CT = 3 and shRNA-ZC3 = 5). (G) Survival curves of mice implanted with shRNA-CT and shRNA-ZC3. (H and I) The graphs show the tumor growth over time (H) and survival curve (I) of mice implanted with HeLa-CT and HeLa-KO-ZC3 (HeLa-CT = 9 and HeLa-KO-ZC3 = 8). Error bars represent SDs, and mean values (tumor volume) were compared using an unpaired two-tailed t test. The log rank test was used to compare the Kaplan-Meier survival curves. ∗*p* < 0.05; ∗∗*p* < 0.01; ∗∗∗∗*p* < 0.0001.

To evaluate the therapeutic potential of ASO-ZC3_1, C57BL/6NRj mice bearing subcutaneous syngeneic B16 melanoma received intratumoral administrations of 10 mg/kg ASO-ZC3H11A for a total of 5 doses on 5 consecutive days (Figure 3A). Notably, by day 17, a marked reduction in tumor volume was observed (Figure 3B), accompanied by enhanced survival rates among treated mice (Figure 3C). The knockdown efficiency was demonstrated on dissected tumors (Figure S6A). Subsequent RNA profiling of the ASO-ZC3_1-treated tumors using NanoString technology revealed distinct alterations in crucial pathways. Significant upregulation was evident in signature scores related to apoptosis, cytotoxicity, Ag presentation, IFN signaling pathways, immune cell migration, and lymphoid and myeloid compartments. Conversely, notable downregulation was observed in cell proliferation pathways (Figure 3D). Moreover, the cell-type scores indicate the presence of more immune cells, including macrophages, DCs, neutrophils, natural killer, and cytotoxic cells in ASO-ZC3_1-treated tumors (Figure 3E). These findings underscore the impact of ASO-ZC3_1 on cancer-related pathways. However, suboptimal delivery might affect the achievement of maximal therapeutic efficacy.

To further address this, we established a stable ZC3H11A knockdown B16 cell line (shRNA-ZC3) using lentivirus-delivered short hairpin RNA (shRNA) (Figures S6B–S6D). The selected cells with stable knockdown of ZC3H11A had a growth rate *in vitro* similar to that of the control cells (Figure S6E), possibly because the selection process excluded the apoptotic cells, and only apoptosis-resistance cells were expanded as stable cell lines. However, when shRNA-ZC3 cells were implanted subcutaneously into mice, we observed a profound delay in tumor growth (Figure 3F) and significantly improved mouse survival (Figure 3G) compared with the control (shRNA-CT). Interestingly, a significantly higher number of CD8^+^ cells infiltrated into shRNA-ZC3 tumors in comparison to the control (shRNA-CT) tumors (Figure S6F), with a similar level of degranulation (Figure S6G).

Finally, to ascertain whether this therapeutic strategy has any potential against human cancers, we engineered human HeLa cells with a ZC3H11A knockout (HeLa-KO-ZC3), followed by tumor growth after injection into athymic nude mice. Slower tumor growth and improved mouse survival were observed in mice with ZC3H11A KO tumors (Figures 3H and 3I) compared to mice that received HeLa-CT (control cell line with Cas9 and nonsense guide RNA). Taken together, these data strongly suggest that ZC3H11A is a valid target for cancer therapy, but the effective targeting of ZC3H11A poses a significant challenge.

## Discussion

According to a recent report, ZC3H11A may be upregulated in cancer cells and may be associated with poor overall survival.^9^ As a component of the TREX complex, ZC3H11A is believed to play a crucial role in RNA processing.^3^^,^^4^^,^^5^^,^^6^ Inappropriate RNA export can lead to the dysregulation of various pathways, contributing to the development of cancer.^14^ This project investigated the impact of ZC3H11A knockdown on cancer pathways and explored its potential as a target for cancer therapy. Our findings indicate that Ag presentation and IFN response are among the highly upregulated pathways. Cancer cells can evade cytotoxic CD8^+^ T cell attack by the downregulation of MHC class I^19^; thus, it is essential to induce Ag presentation through MHC class I to trigger the cytotoxic effects of CD8^+^ cells.^20^ Markedly, the absence of ZC3H11A has been previously reported to be associated with the enrichment of genes involved in the innate immune response, including type I IFN responses.^8^ There is complex interplay between MHC class I and IFN-β. DNA-damaging drugs induce MHC class I expression through the activation of NF-κB signaling.^21^ IFN signaling (including IFN-β) leads to the activation of the NF-κB pathway, inducing the expression of MHC class I on cancer cells.^20^ Interestingly, we also found that the NF-κB pathway is among the upregulated pathways (Figure 1D), as reported previously.^8^ It is likely that knockdown of ZC3H11A induces the expression of MHC class I through the IFN/NF-κB/MHC class I signaling axis.

Zinc finger proteins act primarily as transcription factors involved in various biological processes, including cell proliferation and apoptosis.^22^ It is therefore not surprising that ZC3H11A can play a role in inducing cell proliferation or inhibiting apoptosis. Our results show that ZC3H11A knockdown leads to a decrease in cell viability and the induction of apoptosis, which is considered immunogenic as it enhances CRT exposure and ATP secretion. Furthermore, the functionality of immunogenic apoptosis is demonstrated by increased DC phagocytosis and maturation. Further investigations are needed to know which pathways are targeted by ZC3H11A to inhibit apoptosis and induce the survival of cancer cells.

In our *in vivo* therapeutic experiment utilizing ASO-ZC3_1, we recorded a decrease in tumor growth and an improvement in mouse survival. The RNA profiling of the treated tumor validated our *in vitro* findings, thereby corroborating the efficacy of the ASO. Although we had anticipated more favorable outcomes for tumor growth and mouse survival, the use of a more optimal carrier may have facilitated more accessibility of the ASOs throughout the tumor.^16^^,^^18^^,^^23^ Furthermore, suboptimal release and escape of the ASOs from the endosome could have influenced the results.^24^ To confirm whether ZC3H11A is a viable therapeutic target, we further evaluated tumor growth and survival after subcutaneous injection of the B16 stable cell line with permanent knockdown of ZC3H11A. We observed a marked delay in tumor growth and improved mouse survival. This is in line with the observation when using ASO as therapeutic treatment. Delayed tumor growth is unlikely due to intrinsic cell death since both shRNA-ZC3 and shRNA-CT cells had similar growth kinetics *in vitro*. However, stable knockdown of ZC3H11A led to upregulated MHC class I expression (Figure S6H), increased cytotoxic CD8^+^ cell infiltration, and altered tumor microenvironment. In parallel with the NanoString data, which showed more cytotoxic cells and fewer exhausted CD8^+^ cells. In addition, there were more myeloid compartments, including DCs and macrophages (Figures 3D and 3E). These results strongly indicate that the reduction in tumor growth is most likely attributed to the elicited anticancer immune response. Collectively, these data strongly suggested that ZC3H11A is a valid target for cancer immunotherapy.

One of the limitations of this study is that we cannot exclude the possibility that ASOs targeting ZC3H11A might also affect ZBED6 function (Figures S7A–S7E), a gene located in the first intron of the *ZC3H11A* gene. The expression of these two proteins is controlled by alternative splicing, and if intron retention occurs, ZBED6 will be translated.^2^ However, the mechanism that controls this alternative splicing remains unknown.^25^ ZBED6 is not affected in the CRISPR KO cell line, and the delayed tumor growth of HeLa-KO-ZC3 is a strong indication that specific targeting of ZC3H11A can result in a therapeutic effect. Another thing we cannot rule out is that the designed ASOs (ASO-ZC3_1 and ASO-ZC3_2) might lead to Toll-like receptor (TLR)9 activation, since they incorporate CpG sites in the sequence. However, ASO-ns has two CpG sites and did not show activation of an IFN response. It is reported that two CpG sites can double the activation of the TLR9, but also that the activation could occur in sequences without CpG, and this could be sequence dependent.^26^

Overall, focusing on ZC3H11A in cancer treatment has the potential to hinder tumor growth by boosting the antitumor response. This involves strengthening Ag presentation via MHC class I, promoting the IFN response, and triggering immunogenic apoptosis.

## Materials and methods

### Cell cultures

B16 mouse melanoma cells and human cervical cancer cell line HeLa were cultured in DMEM supplemented with 1% PeSt (penicillin, 100 U/mL, and streptomycin, 100 g/mL), 10% fetal bovine serum (FBS) and 1% sodium pyruvate. HCmel12 is from a primary HGF-CDK4(R24C) melanoma model and is a kind gift from Prof. Thomas Tüting (University Hospital Magdeburg, Germany). CT26 is a mouse colorectal carcinoma cell line (American Type Culture Collection). All the latter cells were cultured in RPMI supplemented with 1% PeSt, 10% FBS, and 1% sodium pyruvate. Media and supplements were purchased from Invitrogen.

### ASOs and *in vitro* transfection

Affinity plus locked nucleic acid (LNA) ASOs targeting mouse ZC3H11A (ASO-ZC3_1, ASO-ZC3_2), as well as ASO-ns were ordered from Integrated DNA Technologies (IDT). The ASOs have phosphorothioate linkages between nucleotides and were modified by the addition of 3 LNA to each end of the ASO (Table S1). Cells at about 75% confluency were transfected with 10 nM ASO, unless stated otherwise. To enhance cellular uptake, ASOs were preincubated with Lipofectamine 3000 (Invitrogen) in serum-free media OPTI-MEM (reduced-serum medium [improved minimal essential medium]) (Invitrogen) for 15 min before adding to the cells. Most assays were performed after 48 h of transfection unless stated otherwise.

### Generation of shRNA-ZC3 cells

For the establishment of B16 cells with sustained knockdown of ZC3H11A (shRNA-ZC3) and control (shRNA-CT), shRNA sequences targeting ZC3H11A gene and nonsense (Figure S6B; Table S1) were ordered from IDT and subcloned into lentiviral vector pBMN(CMV-GLP),^27^ while the shRNA expression was controlled under the H1 promoter. B16 cell lines were then transduced with this lentiviral vector, and stable transduced cells were kept in puromycin selection for 1 week.

### Real-time quantitative PCR (qPCR)

For B16, *ZC3H11A* expression was evaluated using different ASO concentration (0.1, 0.3, 1, 3, and 10 nM) and at different time points (6, 12, 24, and 48 h). For all cell lines, the knockdown efficiency was evaluated at 10 nM after 24 h of transfection. Total RNA was isolated from ASO-transfected cells by using the SingleShot Cell Lysis Kit (Bio-Rad). Reverse transcription and real-time qPCR were performed by using iTaq Universal SYBR Green One-Step Kit (Bio-Rad). The real-time qPCR was done in the CFX96TM Real-Time System (Bio-Rad), and the thermal cycling protocol was applied as described by the manufacturer. The primer sequences are provided in Table S2. The normalized relative expression is analyzed by normalizing to the reference gene (*HPRT*); then, the relative expression of ASO-treated sample to untreated sample was calculated.

### QuantiGene Singleplex expression assay

The QuantiGene Singleplex assay kit and probes targeting mouse *ZC3H11A* and *HPRT* (housekeeping gene) were ordered from Thermo Fisher Scientific. The assay was performed and analyzed by following manufacturer instructions to quantify RNA after transfection with ASOs. This assay is based on branched DNA technology to quantify RNA in the samples.

### Western blot

Protein lysates were prepared from transfected cells using radioimmunoprecipitation assay lysis buffer supplemented with Halt protease and phosphatase inhibitors (Thermo Fisher Scientific). The lysates were denatured for 10 min at 95°C. Proteins in the lysates were separated by 4%–12% Bis-Tris NuPAGE gel (Thermo Fisher Scientific) and transferred to polyvinylidene fluoride membranes (GE Healthcare). Membranes were blocked by 3% BSA and probed with ZC3H11A polyclonal antibody (Ab; 1:500) (Invitrogen), ZBED6 polyclonal Ab (1:100), and anti-β-actin mouse monoclonal Ab (1:500) (Sigma). Membranes were washed with Tris-buffered saline with 0.05% Tween 20 before adding secondary Abs; goat anti-rabbit immunoglobulin G (IgG) (1:2,000) and goat anti-mouse IgG-horseradish peroxidase (1:5,000) (Santa Cruz Biotechnology). Protein bands were visualized using Super Signal West Dura Extended Duration Substrate (Thermo Fisher Scientific). Chemiluminescence was visualized using the iBright CL1500 Imaging System (Thermo Fisher Scientific). Full blots are shown in Figures S8A–S8D.

### NanoString analysis

Total RNA was isolated from ASO-transfected B16 cells using the RNeasy Plus RNA isolation kit (Qiagen). The gene expression levels were directly measured as mRNA counts using the Mouse Tumor Signaling 360 Panel (NanoString). Analysis of gene expression was performed using nSolver Analysis software (NanoString). The pathways scores are available in Tables S3 and S4.

### Flow cytometry analysis

Cells were washed with PBS supplemented with 3 mM EDTA (Thermo Fisher Scientific) and stained with fluorescence-labeled Abs according to the specific experiment. The stained cells were washed with PBS supplemented with 3 mM EDTA and analyzed with CytoFLEX LX flow cytometer (Beckman Coulter) and FlowJo software (FlowJo LLC). The gating strategy is shown in Figures S9A–S9C and S10A–S10C.

### Detection of MHC class I expression

MHC class I expression was detected by flow cytometry after staining with Abs. FITC anti-mouse H-2D(b) (1:200) (BD Biosciences) for B16 and Hcmel12 and APC anti-mouse H-2Kd/H-2Dd (1:200) for CT26.

### Detection of IFN-β

The cells were seeded and transfected with ASOs in 48-well plates. After 24 h of transfection, the cells were treated with polyI:C (50 μg/mL) for another 24 h. Supernatants were collected for detection of released mouse IFN-β using ELISA (DuoSet ELISA, R&D Systems).

### Cell viability assays

The cells were seeded and transfected by ASOs in 96-well plates. The viability was analyzed by CellTiter 96 AQueous One Solution Cell Proliferation Assay (MTS) (Promega) after 24, 48, and 72 h of transfection. In another experimental setting using 48-well plates, the transfected cells were collected and stained by 1.5 μM ethidium homodimer-1 (EthD-1) (Invitrogen). The stained dead cells were analyzed by flow cytometry. shRNA-CT-B16 and shRNA-ZC3-B16 (1 × 10^5^ cells/well) were seeded in xCELLigence E-plate 16 (Agilent) and incubated at 37°C. Cell viability was tracked in real time by measuring the cell index using xCELLigence RTCA SP (ACEA Biosciences).

### Cell apoptosis assays

Cells were seeded and transfected by ASOs in 96-well plates. Caspase activity was measured using the Caspase-Glo 3/7 Assay System (Promega). In another experimental setting in 48-well plates, the ASO-transfected cells were stained with APC-annexin V (1:50) (BioLegend) and 7-aminoactinomycin D (7AAD) (1:50) (BD Biosciences) and analyzed by flow cytometry.

### Immunogenic apoptosis assays

For the detection of CRT exposure, the cells were stained after 36 h of ASO transfection with the anti-CRT antibody (1:50) (catalog no. PA3-900, Thermo Fisher Scientific) followed by secondary donkey anti-rabbit IgG-Alexa Fluor 488 (2 μg/mL) (Thermo Fisher Scientific) and 7AAD (1:50) (BD Biosciences). The analysis was performed by flow cytometry. Extracellular ATP was detected in the supernatant of ASO-treated cells by using CellTiter-Glo 2.0.

### Isolation of mouse bone marrow-derived DCs

The Northern Stockholm Research Animal Ethics Committee (5.8.18-19434-2019) approved the animal studies, which were performed at Uppsala University. Bone marrow cells were obtained from the femurs and tibias of C57BL/6NRj mice (H-2Db) and cultured in IMDM (Iscove’s modified Dulbecco’s medium) supplemented with 1% PeSt, 10% FBS, 1 mM HEPES, and 50 μM β-mercaptoethanol. Media and supplements were purchased from Invitrogen. The media was also supplemented with recombinant murine interleukin-4 (20 ng/mL) and recombinant murine granulocyte-monocyte-colony-stimulating factor) (20 ng/mL) from Nordic BioSite. The cells were plated on non-treated Petri dishes (Sarstedt), and the media were changed every 3 days. The non-adherent imDCs were collected on day 7.

### Functional immunological assay

For DC phagocytosis, ASO-treated cells were stained with CellTrace violet dye (CTV, 5 μg/mL) (Thermo Fisher Scientific) and co-cultured with imDCs for 2 h. The CTV + DCs were considered phagocytic cells and were quantified by flow cytometry after gating for CD11c^+^ (phycoerythrin [PE] anti-mouse CD11c [BioLegend]). For DC maturation, imDCs were cocultured with ASO-treated cells for 24 h. The DC maturation markers were analyzed by flow cytometry after staining with the following Abs: PE anti-mouse CD11c (BioLegend), BB515 rat anti-CD11b (BD Biosciences), Brilliant Violet 510TM anti-mouse I-A/I-E (BioLegend), PE/Cy5 anti-mouse CD40 (BioLegend), and APC anti-mouse CD86 (BioLegend), all prepared with a dilution of 1:200.

### *In vivo* experiment

Ethical permits (N164/15 and 5.8.18-19434-2019) were approved by the Northern Stockholm Research Animal Ethics Committee. *In vivo* experiments were performed at Uppsala University with 8-week-old female C57BL/6NRj mice (H-2Db) or athymic nude (ATHYM-Foxn1^nu/nu^).

The C57BL/6NRj mice were injected subcutaneously in the right hind flank with B16 or shRNA-ZC3 or shRNA-CT (1 × 10^5^ cells/100 μL PBS). HeLa-CT or HeLa-KO-ZC3 were generated as described previously^6^ using CRISPR-Cas9 technology, and 1 × 10^6^ cells/100 μL PBS were injected subcutaneously into athymic nude mice. For the therapeutic experiment, 25 μL ASOs (10 mg/kg) preincubated with PEI (polyethyleneimine) was administered intratumorally to the subcutaneous B16 melanoma tumors of the mice. The ASO treatment was performed five times in total (consecutive days). When the tumors reached the humane endpoint of 1,000 or 1,500 mm^3^, due to varying ethical permits used for different studies, the mice were euthanized. The tumor size was calculated using the formula ume = length × width^2^ × π/6. The time-to-endpoint (TTE) for each mouse was calculated using the formula: TTE = [log (EPV)–*b*]/*m*, where *b* is the constant of the intercept and *m* is the slope of the line obtained by linear regression (time vs. tumor volume) of a log-transformed tumor growth dataset, which comprised four consecutive measurements before the endpoint value (EPV). TTE value is the day of death when the animal is determined to have died due to treatment-related causes. TTE values were used to generate the Kaplan-Meier survival curve and compared using the log rank (Mantel-Cox) test. All tumor measurements are provided as supplementary tables (Tables S4–S6).

### *In vivo* NanoString analysis

After establishment of the therapeutic experiment, we injected ASO as stated before a total of three times, and the tumors were dissected 3 days after the last injection. Total RNA was isolated using the RNeasy Plus RNA isolation kit (Qiagen). The Mouse-PanCancer Immuno-Oncology Kit (NanoString) was used to determine gene expression levels by directly counting mRNA. nSolver Analysis software (NanoString) was used to perform gene expression analysis. All pathways and cell-type score (transformed *Z* score) are available in Tables S7 and S8.

### *In vivo* flow cytometry analysis

After the dissection of tumors from the mice implanted with shRNA-ZC3 or shRNA-CT, the tumors were mashed to obtain single cells. Then, the cells were stained with BV421 anti-mouse CD3 (BioLegend), APC-Cy7 anti-mouse CD8 (BioLegend), and PE anti-mouse CD107a (BioLegend) at a dilution of 1:50 for all Abs. The stained cells were washed with PBS supplemented with 3 mM EDTA and analyzed with the BD Canto II flow cytometer (BD Biosciences) and FlowJo software. The gating strategy is shown in Figure S11A.

### Statistical analysis

GraphPad Prism software version 10 was used to perform the statistical analysis. The data are reported as means ± SDs. The mean values of the two groups were compared through an unpaired two-tailed t test. The log rank test was used to compare Kaplan-Meier survival curves. Values with *p* < 0.05 were considered to be statistically significant.

## Data and code availability

Data are accessible upon request from the authors.

## Acknowledgments

This research was supported by the 10.13039/501100004063Knut and Alice Wallenberg Foundation (KAW 2020.0211, to L.A. and M.E.), the 10.13039/501100002794Swedish Cancer Society (22 2229 Pj, to D.Y.), the Swedish Children Cancer Society (PR2022-0105, to D.Y.), the 10.13039/501100004359Swedish Research Council (ME, 2023-02232), the 10.13039/501100002794Swedish Cancer Society (22 2241 Pj, to M.E.), the Swedish Childhood Cancer Fund (PR2023-0103, to M.E.). We would like to thank KIGene Annika Eriksson at Karolinska University Hospital for help on the NanoString assay readout. We would also like to thank the BioVis Platform at Uppsala University for help with flow cytometry. It is also important to thank Tanel Punga for giving us the ZBED6 antibody. Many thanks to Shady Younis who provide the modified HeLa cell lines.

## Author contributions

A.A., M.E., L.A., C.J., and D.Y. designed the experiments and conceived the study. A.A., P.C., M.D., and C.J. performed the experiments and analyzed the data. A.A. wrote the first draft manuscript, and all authors revised the paper. All authors approved the final version of the manuscript.

## Declaration of interests

The authors declare no competing interests.

## Supplemental information

Data S1. Tables S3–S8

Document S2. Article plus supplemental information
