# Supplementary material for: Targeting ZC3H11A elicits immunogenic cancer cell death through augmentation of antigen presentation and interferon response
Source: Mol Ther Nucleic Acids. 2024 Oct 21;35(4):102361. doi: 10.1016/j.omtn.2024.102361 (PMC11585804; doi:10.1016/j.omtn.2024.102361)
Supplement: Document S1. Figures S1–S11 and Tables S1 and S2 [file mmc1.pdf]

OMTN, Volume *35*

Supplemental information

Targeting ZC3H11A elicits immunogenic cancer cell death through augmentation of antigen presentation and interferon response

Arwa Ali, Paola Contreras, Mahmoud Darweesh, Leif Andersson, Chuan Jin, Magnus Essand, and Di Yu

Figure S1

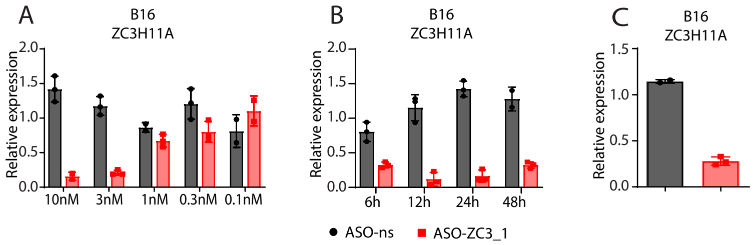

Figure S1. Knockdown efficiency of ASO-ZC3_1 in B16 cells. (**A**) The Knockdown efficiency of ASO-ZC3_1 at different concentrations (10, 3, 1, 0.3 and 0.1nM) and (**B**) different time points (6, 12, 24 and 48h) at RNA level in B16 cells by RT-qPCR. The expression of ZC3H11A gene was normalized to HPRT (reference gene) and relatively expressed to untreated cells (only media) (**C**) The Knockdown efficiency of ASO-ZC3_1 in B16 at 10nM assessed by QuantiGene Singleplex assay. ZC3H11A gene expression was normalized to HPRT and fold change to untreated (only media) cells was depicted in the graph (n= 2-3 replicates/group).

Figure S2

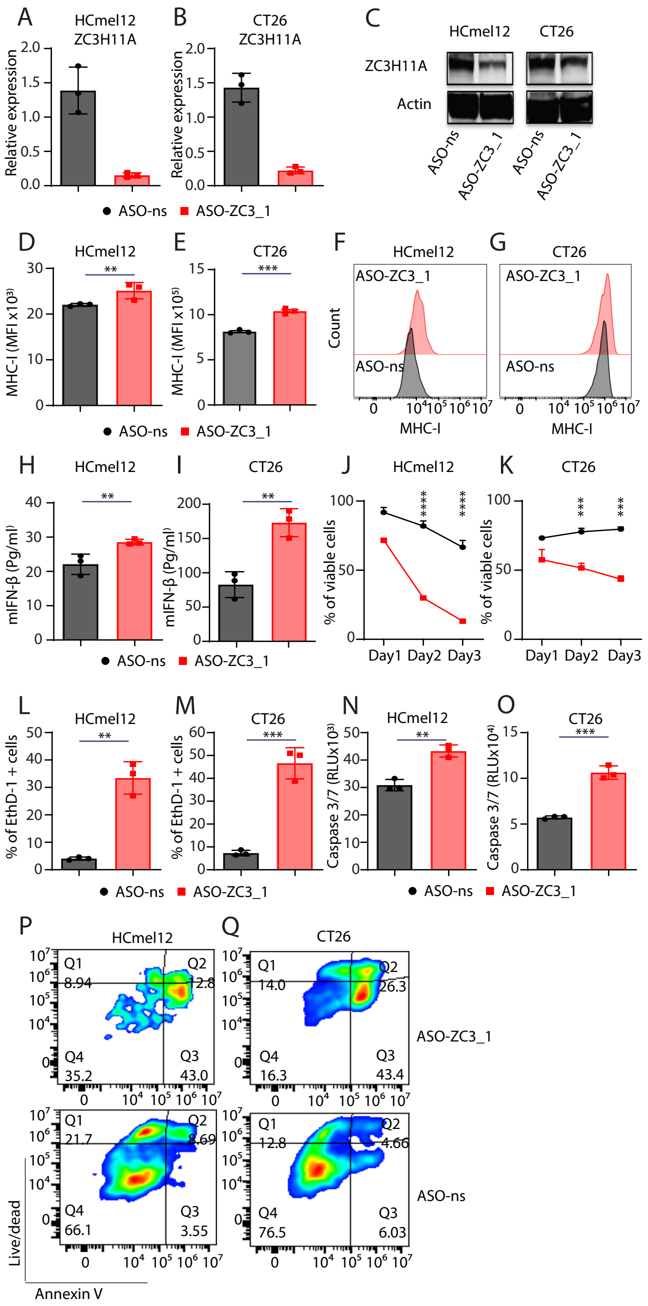

Figure S2. Knockdown of ZC3H11A by ASO-ZC3_1 enhanced antigen presentation, IFN response and apoptosis in HCmel12 (mouse melanoma) and CT26 (mouse colorectal carcinoma) cells. (**A and B**) The Knockdown efficiency of ASO-ZC3_1 (10nM) estimated at RNA level of HCmel12 and CT26 by RT-qPCR, ZC3H11A gene expression was normalized to HPRT and relative expression was calculated to untreated cells (only media) (**C**) western blot shows knockdown efficiency of ASO-ZC3_1 at protein level by in HCmel12 and CT26, Actin used as reference protein. (**D-G**) The expression of MHC-I in HCmel12 and CT26 after ASO treatment by flow cytometry with representative histograms. (**H and I**) The ELISA analysis of IFN-β released in supernatants after ASO transfection and polyI:C (50 µg/ml) treatment of HCmel12 and CT26. (**J and K**) line graphs show the viable cells percentages of both ASO-ns and ASO-ZC3_1 transfected HCmel12 and CT26 over 3 consecutive days. (**L and M**) The percentage of EthD-1+ cells (dead cells) after ASO treatment of HCmel12 and CT26. (**N and O**) The Caspase3/7 level (RLU) for both cell lines after ASO treatment. (**P and Q**) the representative plots showing the percentages annexin v+ cells in both HCmel12 and CT26 after ASO treatment. (n= 3 replicates/group). Error bars represent SD, and the mean values were compared using an unpaired two-tailed T-test. Key to statistics: **: P<0.01, ***: P<0.001.

Figure S3

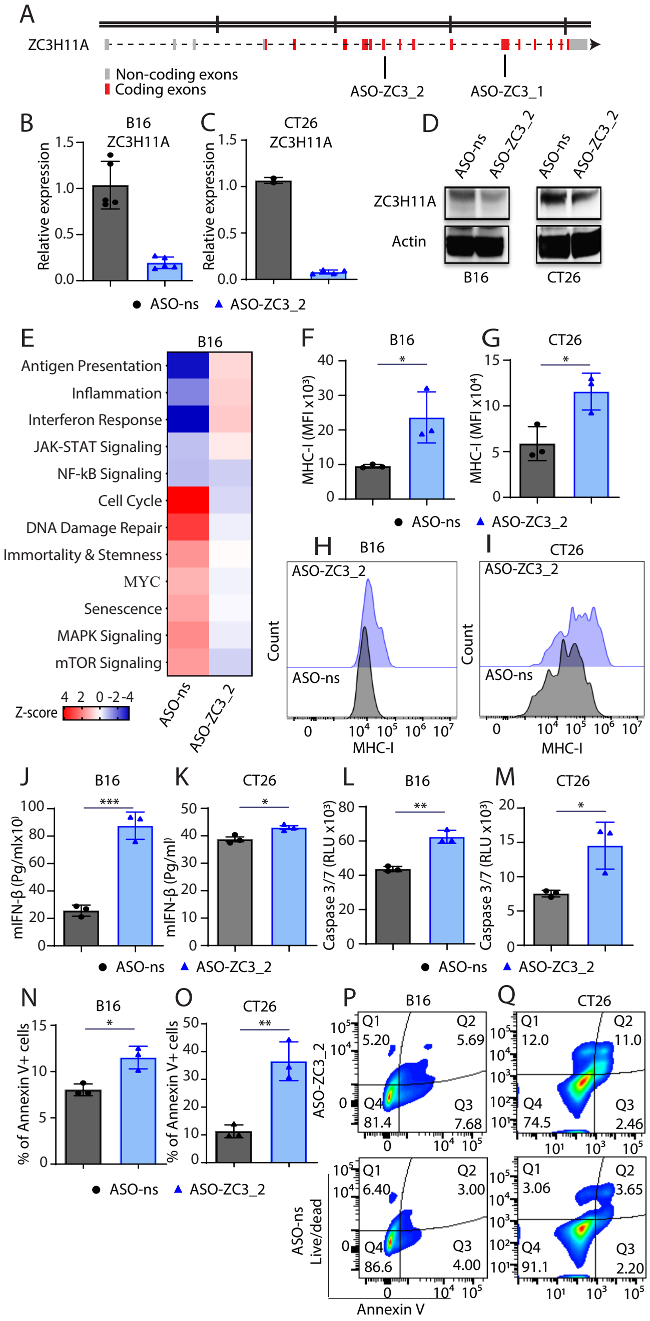

Figure S3. ASO-ZC3_2 results in enhancing antigen presentation, IFN response and immunogenic apoptosis in B16 and CT26. (**A**) Schematic illustration describing the location ASO-ZC3_2 target within ZC3H11A gene. (**B and C**) The Knockdown efficiency of ASO-ZC3_2 (10nM) at RNA level by RT-qPCR for both B16 and CT26, the expression of ZC3H11A gene was normalized to the refence gene (HPRT) and relative expression was normalized to untreated cell. (**D**) Western blot shows knockdown efficiency of ASO-ZC3_2 (10nM) at protein level in B16 and CT26. (**E**) The heatmap shows NanoString signature scores of the different pathways that were upregulated or downregulated after treatment with ASO-ns and ASO-ZC3_2 in B16 cell. (**F -I**) The expression of MHC-I by flow cytometry with representative histograms for both B16 and CT26 after ASO treatment. (**J and K**) The ELISA analysis of IFN-β released in supernatants of ASOs transfected B16 and CT26 that treated with polyI:C (50 µg/ml). (**L and M**) The Caspase3/7 level (RLU) of B16 and CT26 after treatment with ASO. (**N-Q**) The percentages of annexin v+ cells with representative plots. (n= 3 replicates/group). Error bars represent SD, and the mean values were compared using an unpaired two-tailed T-test. Key to statistics: *: P<0.05, **: P<0.01***: P<0.001.

Figure S4

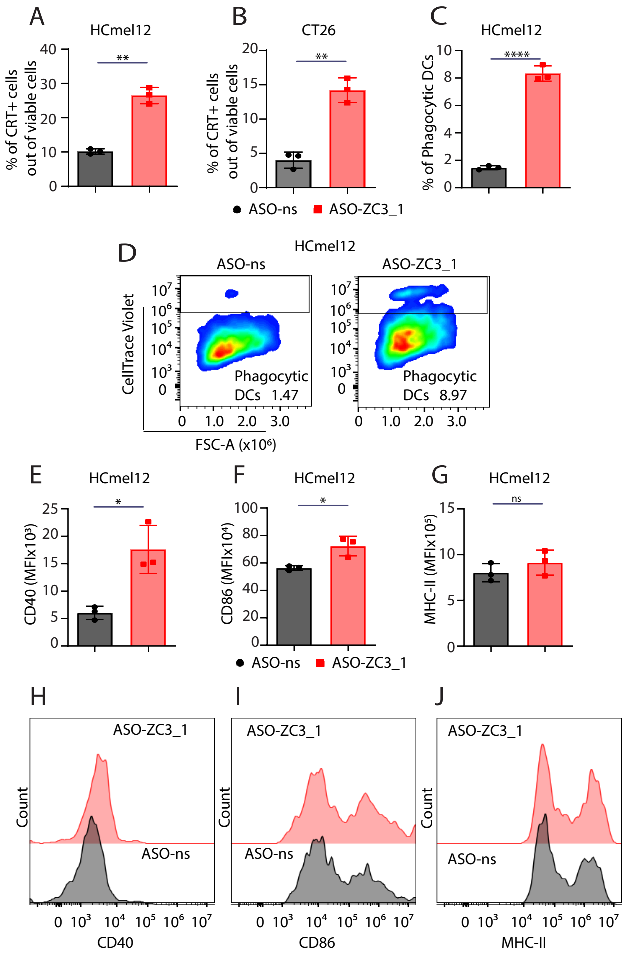

Figure S4. ASO-ZC3_1 induces immunogenic apoptosis in HCmel12 and CT26. The percentage of calreticulin (CRT)+ cells analyzed out of viable cells by flow cytometry in (**A**) HCmel12 and (**B**) CT26 after 36h of ASO transfection. (**C and D**) The percentage of dendritic cells (DCs) that phagocytized ASOs transfected HCmel12 which stained with CellTrace violet (CTV) and representative plots of flow cytometry. Mean fluorescent intensity (MFI) of DCs activation and maturation markers (**E**) CD40, (**F**) CD86 and (**G**) MHC-II after co-culturing DCs with ASOs transfected HCmel12 and (**H-J**) representative histograms. (n= 3 replicates/group). Error bars represent SD, and the mean values were compared using an unpaired two-tailed T-test. Key to statistics: ns: nonsignificant, $P>0.05$, *: $P<0.05$, **: $P<0.01$, ****: $P<0.0001$.

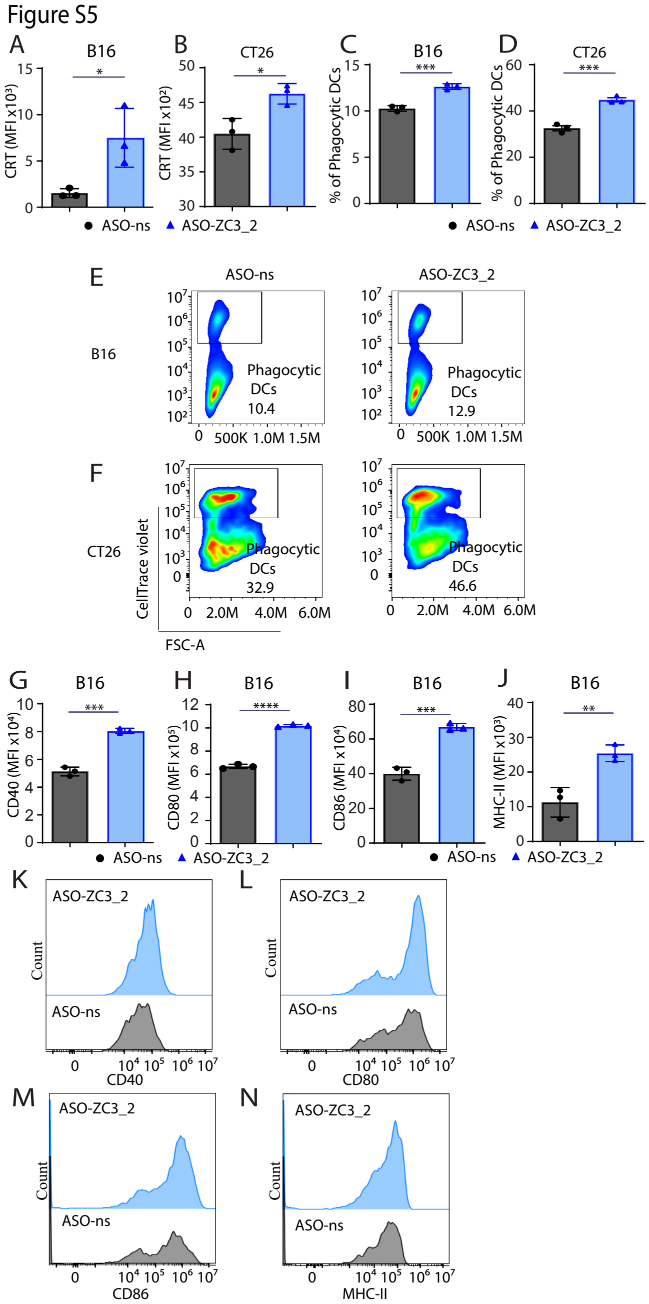

Figure S5. ASO-ZC3_2 induces immunogenic apoptosis in B16 and CT26. (**A and B**) The Mean fluorescent intensity (MFI) of calreticulin (CRT) analyzed by flow cytometry for viable cells for both B16 and CT26 that treated with ASOs for 36h. (**C and D**) The percentage of phagocytic dendritic cells (DCs) after co-culturing with ASOs transfected B16 and CT26 that stained with CellTrace violet, (**E and F**) are representative flow cytometry plots. MFI of DCs activation and maturation markers (**G**) CD40, (**H**) CD80 (**I**) CD86 and (**J**) MHC-II after co-culturing with ASOs transfected B16 with representative histograms (**K-N**). (n= 3 replicates/group). Error bars represent SD, and the mean values were compared using an unpaired two-tailed T-test. Key to statistics: *: P<0.05, **: P<0.01***: P<0.001, ****: P<0.0001.

Figure S6

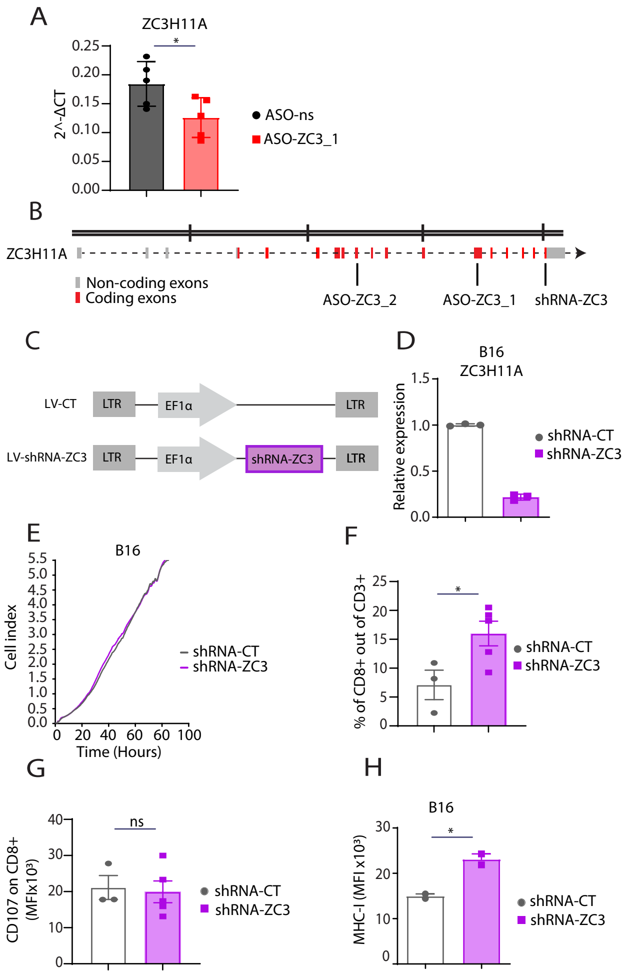

Figure S6. Targeting mouse ZC3H11A using different strategies. (**A**) The relative expression ($2^{-\Delta CT}$) of ZC3H11A in dissected tumor after treatment with ASO-ns and ASO-ZC3_1. The $2^{-\Delta CT}$ value was calculated after normalization to HPRT (reference gene) (n= 5 mice/group) (**B**) Schematic illustration showing locations of different ASOs (ASO-ZC3_1 and ASO-ZC3_2), shRNA and gRNA targets within murine ZC3H11A gene. (**C**) Illustration of shRNA constructs incorporated in lentivirus that used to generate shRNT-CT-B16 and shRNA-ZC3-B16 cell line. (**D**) The knockdown efficiency of B16 cell line after treatment with lentivirus with shRNA targeting ZC3H11A by RT-qPCR, the expression of ZC3H11A was normalized to HPRT and the relative expression calculated to un treated (wild type) B16 (n= 3 replicates/group). (**E**) The growth of both shRNA-CT-B16 and shRNA-ZC3-B16 cells over time using xCELLigence RTCA. (**F**) The percentages of CD8+ cells out of CD3+ cells, and (**G**) MFI of CD107 on CD8+ cells in shRNA-CT-B16 and shRNA-ZC3-B16 dissected tumors (n= 3-5 mice/group). (**H**) The MFI of MHC-I on shRNA-CT-B16 and shRNA-ZC3-B16 cells (n= 2 replicates/group). Error bars represent SD, and the mean values were compared using an unpaired two-tailed T-test. Key to statistics: ns: nonsignificant, *: P<0.05, **: P<0.01***: P<0.001, ****: P<0.0001.

Figure S7

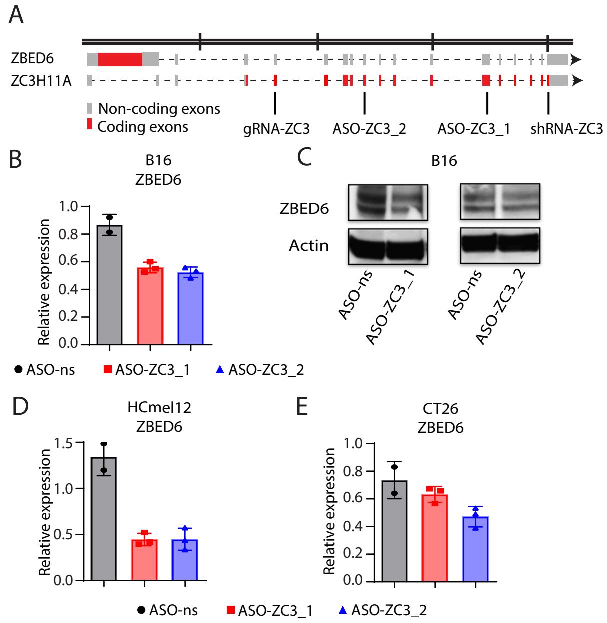

Figure S7. The effect of ASO targeting ZC3H11A on the expression of ZBED6. (A) Schematic illustration showing the location of ASO-ZC3_1 and ASO-ZC3_2 targets within ZC3H11A and ZBED6 genes. (**B**) The relative expression of ZBED6 (RT-qPCR) to untreated cells was calculated after normalization to HPRT as a reference gene of B16 transfected with ASO-ZC3_1 and ASO-ZC3_2 (**C**) Immunoblots shows protein level in B16 cells after treatment with ASO-ZC3_1 and ASO-ZC3_2. (**D and E**) The relative expression of ZBED6 at RNA level (RT-qPCR) on HCmel12 and CT26 (n= 2-3 replicates/group).

Figure S8

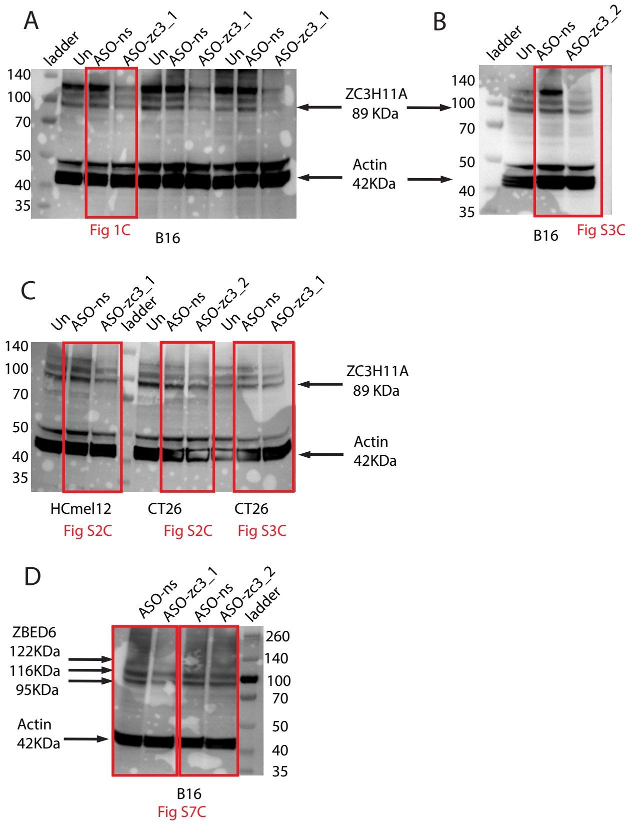

Figure S8. Protein level of ZC3H11A and ZBED6 after ASOs treatment. Immunoblots show ZC3H11A (89KDa) and reference protein (Actin 42KDa) bands in untreated (un), ASO-ns, ASO-ZC3_1 (3 replicates) and ASO-ZC3_2 treated B16 (**A and B**), HCmel12 and CT26 cells (**C**). (**D**) ZBED6 bands (95,116, 122KDa) in B16 cells after ASO-ns, ASO-ZC3_1 and ASO-ZC3_2 treatment. The red highlights indicate the corresponding plots shown in Fig 1C, Fig S2C, Fig S3C and Fig S7C.

Figure S9

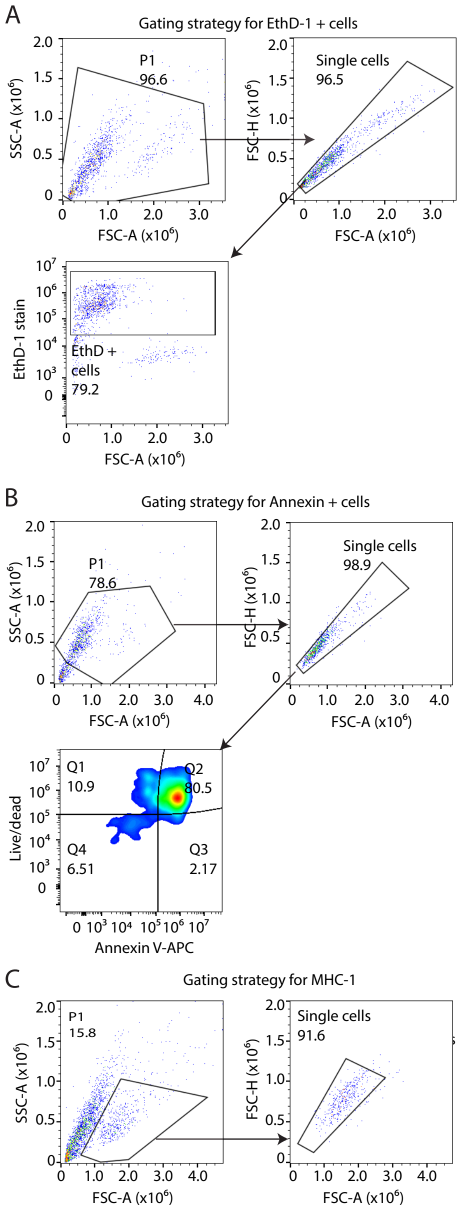

Figure S9. Flow cytometry gating strategies. The figure illustrates the gating strategies used in flow cytometry to analyze (**A**) EthD-1 + cells out of singlets (**B**) Annexin V-APC against Live/dead stain (7AAD) to identify Annexin V-positive cells, and (**C**) mean fluorescence intensity (MFI) of MHC-I on single cells.

Figure S10

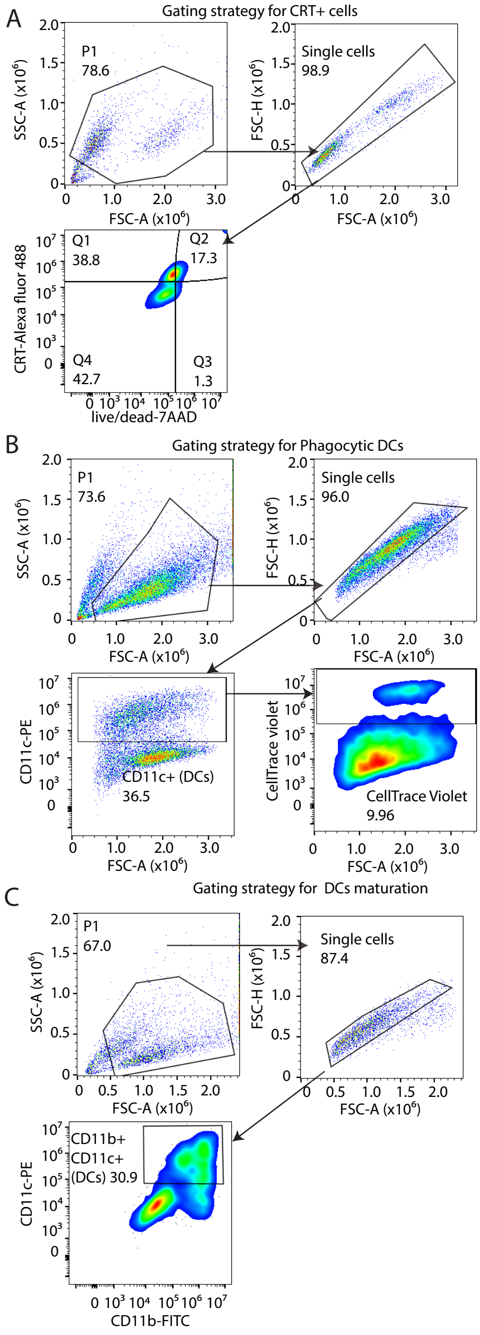

Figure S10. Strategies for gating flow cytometry plots. (**A**) The figure depicts the gating strategies employed to determine CRT (Calreticulin) expression on viable cells. (**B**) Phagocytic dendritic cells (DCs) were gated based on CD11C+ and CellTrace violet (CTV) positivity among single cells. (**C**) MFI analysis for various DC maturation markers was applied on CD11b+ CD11c+ cells.

Figure S11

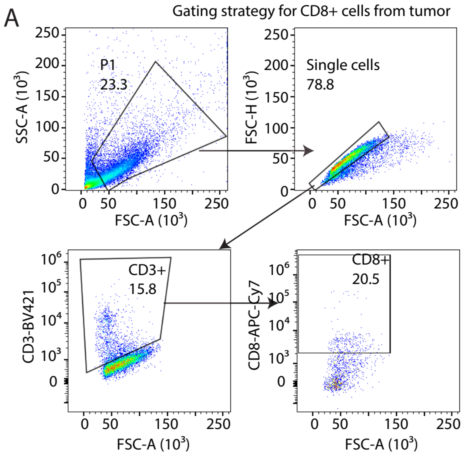

Figure S11. Strategy for gating CD8+ cells from dissected tumor. (**A**) The figure shows the gating strategy used to determine the percentages of CD8+ out of CD3+ cells and MFI of CD107 (degranulation marker) was gained from CD8+ population.

Table S1: Sequences of antisense oligonucleotides (ASOs), and shRNA

**ASO**	**Sequence**
ASO-ns	+G*+A*+C*T*A*T*A*C*G*C*G*C*A*+A*+T*+A
ASO-ZC3_1	+G*+C*+A*A*T*T*C*T*C*C*A*C*G*+T*+T*+T
ASO-ZC3_2	+T*+C*+C*G*A*A*C*A*T*T*C*T*C*+C*+T*+T
shRNA-ns	GCGCGATAGCGCTAATAATTT
shRNA-ZC3	AAGTCGATCTCAGCTTCCAA

+= locked nucleic acid base
*= Phosphorothioate bond

Table S2: Sequences of Primers

**Gene**	**Primer sequence**
ZC3H11A-F	TCAGTGGGTGGTGATAGTGAC
ZC3H11A-R	TCCACGTTTCTGACTGGCTC
HPRT-F	CAAACTTTGCTTTCCCTGGT
HPRT-R	TCGAGAGGTCCTTTTCACC
ZBED6-F	CAAGACATCTGCAGTTTGGAATTT
ZBED6-R	TGTCGTTGAAGTGTTGAAGTTCCTA

Table S3: Pathway Scores (transformed Z scores) of invitro Nanostring data
Table S4: Tumor size over time for mouse B16 tumor treated with ASO-ns or ASO-ZC3
Table S5: Tumor size over time of B16-shRNA-CT and B16-shRNA-ZC3
Table S6: Tumor size over time of HeLa-CT and HeLa-KO-ZC3
Table S7: Pathway Scores (transformed Z scores) of invivo Nanostring data
Table S8: Cell type Scores (transformed Z scores) of invivo Nanostring data
